# A Challenging Diagnosis of Cushing’s Syndrome in Primary Care: A Case Report

**DOI:** 10.7759/cureus.108265

**Published:** 2026-05-04

**Authors:** Mariana Francisco Ferreira, Fábio Valente, Rosa Correia, Pedro Apolinário

**Affiliations:** 1 Family Medicine, USF Fénix, ULS Trás-os-Montes e Alto Douro, Vila Real, PRT

**Keywords:** acth-independent hypercortisolism, adrenal adenoma, cushing syndrome, functional impairment, musculoskeletal pain, primary care

## Abstract

Cushing’s syndrome is a rare endocrine disorder characterized by an insidious course and multisystem manifestations, often leading to delayed diagnosis due to its nonspecific presentation. A 52-year-old woman with a history of type 2 diabetes mellitus and hypertension was followed in primary care for persistent musculoskeletal pain and worsening metabolic control, initially attributed to poor treatment adherence. Her condition progressively led to significant functional impairment, requiring the use of crutches for ambulation and ultimately resulting in medical retirement due to disability. After presenting with abdominal pain, a computed tomography scan incidentally revealed a 35 mm left adrenal mass suggestive of an adenoma. In light of the clinical context, further evaluation confirmed adrenocorticotropic hormone (ACTH)-independent hypercortisolism. The patient underwent left adrenalectomy and remains under clinical follow-up, with progressive improvement in metabolic parameters and functional status. This case highlights the importance of reassessing persistent symptoms and appropriately evaluating incidental findings in primary care, as well as the potential for significant functional decline in undiagnosed cases. Early recognition of atypical presentations may facilitate the diagnosis of rare endocrine disorders.

## Introduction

Cushing’s syndrome is a rare endocrine disorder caused by prolonged exposure to excessive cortisol levels. Its clinical presentation is often insidious and characterized by multisystem involvement, including arterial hypertension, diabetes mellitus, cutaneous changes, osteoporosis, and muscle weakness. These manifestations are frequently nonspecific, contributing to delayed diagnosis and underrecognition by healthcare professionals [1,2].

In primary care, a patient-centered approach combined with longitudinal follow-up allows the identification of progressive and persistent changes, as well as the functional impact of symptoms. This case illustrates the role of the family physician in reassessing persistent complaints and integrating incidental findings, ultimately leading to the diagnosis of adrenocorticotropic hormone (ACTH)-independent hypercortisolism of adrenal origin.

## Case presentation

A 52-year-old female factory worker with a history of type 2 diabetes mellitus and arterial hypertension was regularly followed in a primary care setting. She repeatedly reported generalized musculoskeletal pain and progressive functional decline over several years.

Despite evaluation by multiple hospital specialties and prolonged physiotherapy, she maintained significant pain complaints with increasing limitation of daily activities. Glycated hemoglobin (HbA1c) was 8.6%, later 7.8% (reference: 4.0-5.6%) after therapeutic adjustment. Blood pressure was 170/90 mmHg, subsequently 153/100 mmHg. Lipid profile showed total cholesterol 264 mg/dL (reference: <200 mg/dL), low-density lipoprotein (LDL) cholesterol 175 mg/dL (reference: <130 mg/dL), and triglycerides 212 mg/dL (reference: <150 mg/dL). Despite treatment intensification, these parameters remained above target.

In parallel, her functional status progressively worsened, requiring assistance for ambulation or the use of crutches. This decline ultimately led to medical retirement due to disability.

**Figure 1:**
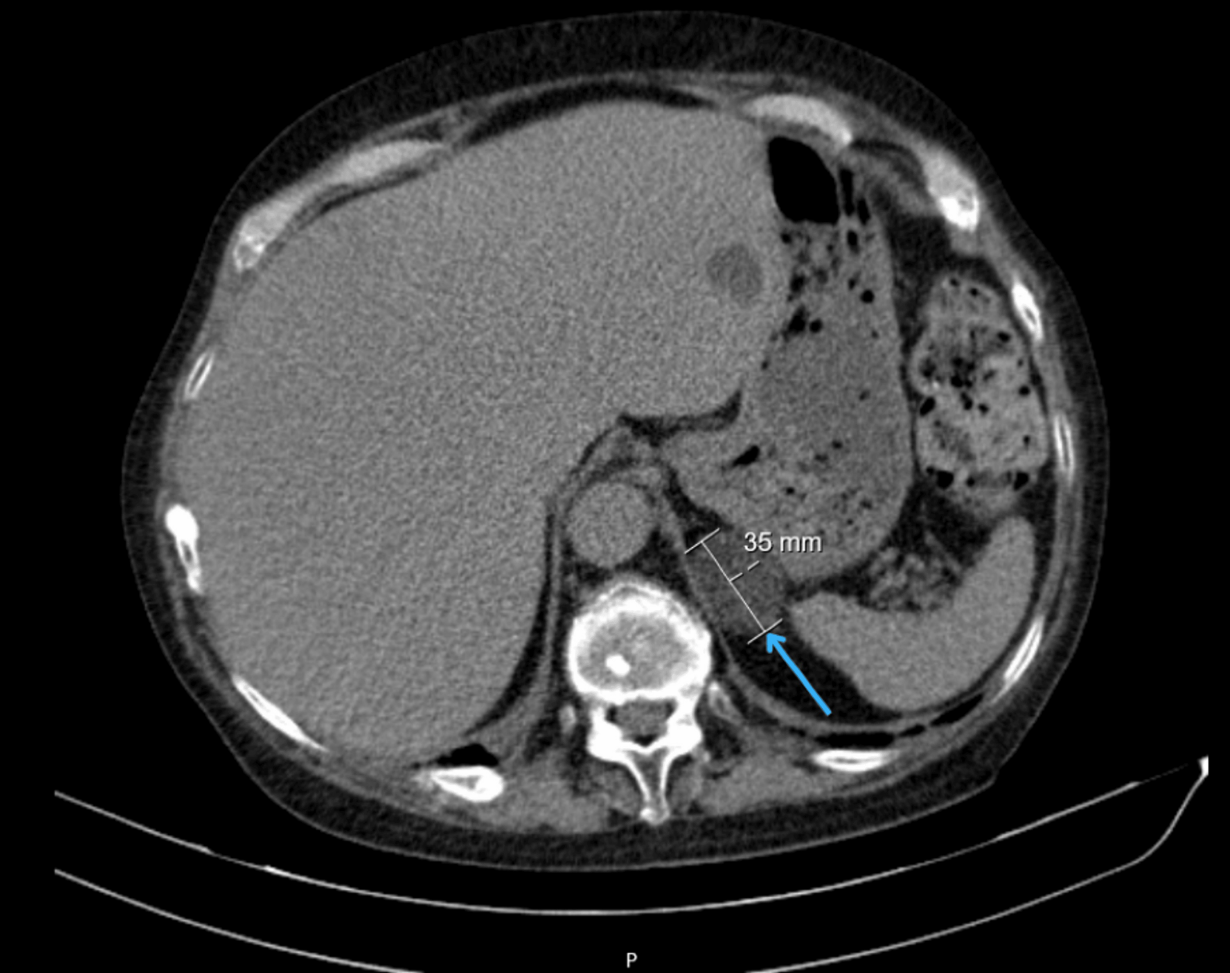
Abdominal computed tomography scan showing left adrenal mass Axial abdominal CT image demonstrating a 35 mm solid nodular lesion in the left adrenal gland (arrow), consistent with an adrenal adenoma.

Approximately three years after symptom onset, she presented to a private hospital emergency department with a six-month history of painful swelling in the left hypochondrium. Abdominal computed tomography revealed a 35 mm solid nodular lesion in the left adrenal gland, suggestive of an adenoma (Figure 1).

**Figure 2:**
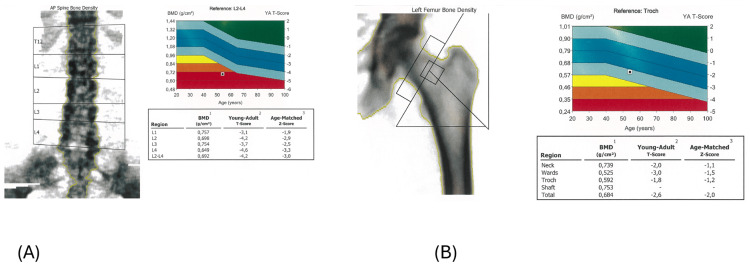
Bone densitometry showing severe osteoporosis (A) Lumbar spine dual-energy X-ray absorptiometry (DEXA) scan demonstrating severe osteoporosis (T-score −4.2). (B) Femoral DEXA scan showing reduced bone mineral density (T-score −2.6).

At a subsequent primary care consultation, she presented with moon facies, facial plethora, proximal muscle atrophy, and scattered ecchymoses. Bone densitometry revealed severe osteoporosis (lumbar spine T-score −4.2, femoral T-score −2.6) (Figure 2) [3,4].

Laboratory studies showed elevated serum cortisol (727 nmol/L; reference: 171-536 nmol/L) with suppressed morning ACTH (1.5 ng/L; reference: 7.2-63.3 ng/L). Based on these findings, she was referred to endocrinology. In the endocrinology department, a 1 mg overnight dexamethasone suppression test showed cortisol of 637.24 nmol/L (reference: 171-536 nmol/L), confirming ACTH-independent hypercortisolism [1,2].

She subsequently underwent laparoscopic transperitoneal left adrenalectomy without complications. Histopathological examination confirmed an adrenocortical adenoma, showing a well-circumscribed lesion with diffuse architecture and cortical atrophy of the surrounding parenchyma. No evidence of necrosis, vascular invasion, or capsular invasion was identified. Immunohistochemical analysis demonstrated CYP11B1 positivity and CYP11B2 negativity, consistent with a cortisol-producing adenoma.

Following surgery, the patient remained under follow-up, with progressive improvement in metabolic parameters and functional status.

**Figure 3:**
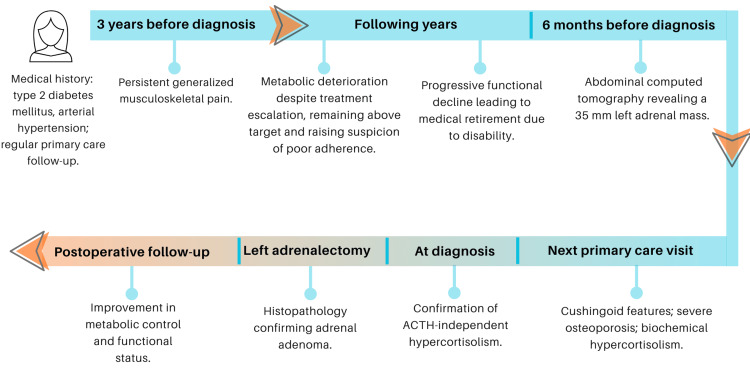
Timeline of clinical events, investigations and management. ACTH: adrenocorticotropic hormone. Created by the authors using Canva (Sydney, Australia).

A timeline summarizing the clinical course, investigations, and management is presented in Figure 3.

## Discussion

Cushing’s syndrome is a rare condition, with an estimated incidence of two to three cases per million people per year [1]. Its clinical presentation is variable and often masked by common comorbidities such as diabetes mellitus and hypertension, which may delay diagnosis [2]. This overlap frequently contributes to diagnostic delay, often extending over several years [1,2].

Adrenal incidentalomas are increasingly identified due to widespread use of imaging, and their evaluation requires careful hormonal assessment to exclude functional lesions [5]. In this case, the incidental finding of an adrenal mass, combined with clinical suspicion, prompted further investigation and ultimately led to diagnosis. The progressive and multisystem nature of hypercortisolism often results in significant morbidity, including severe osteoporosis and functional decline, as observed in this patient [3]. Chronic exposure to excess cortisol is known to negatively impact bone metabolism and increase fracture risk [3].

The 1 mg overnight dexamethasone suppression test is widely used as an initial screening tool, with a cortisol cut-off of <1.8 μg/dL providing high sensitivity for the diagnosis of Cushing’s syndrome [1,2].

The holistic and continuous approach of the family physician allows recognition of subtle clinical changes and the functional impact of symptoms. This role is particularly relevant in slowly progressive and multisystem diseases such as Cushing’s syndrome.

It is important to note that patients with frequent consultations and persistent complaints may have their concerns undervalued. The tendency to attribute such presentations to anxiety, low pain tolerance, or poor adherence is understandable in a high-demand clinical setting but may contribute to delayed diagnosis of serious conditions.

## Conclusions

This case highlights the importance of the family physician in recognizing and valuing persistent symptoms and their functional impact. The longitudinal follow-up was essential in raising suspicion and guiding diagnosis. In patients with repeated complaints, careful clinical assessment is crucial to avoid underestimation of potentially serious conditions. Particular attention should be given to unexplained functional decline, even when symptoms appear nonspecific. Maintaining clinical vigilance is fundamental to improving patient outcomes.
